# Mitotic read-out genes confer poor outcome in luminal A breast cancer tumors

**DOI:** 10.18632/oncotarget.15562

**Published:** 2017-02-21

**Authors:** Javier Pérez-Peña, Ana Alcaraz-Sanabria, Cristina Nieto-Jiménez, Raquel Páez, Verónica Corrales-Sánchez, Leticia Serrano-Oviedo, Vikram B. Wali, Gauri A. Patwardhan, Eitan Amir, Balázs Győrffy, Atanasio Pandiella, Alberto Ocaña

**Affiliations:** ^1^ Translational Research Unit, Albacete University Hospital and Centro Regional de Investigaciones Biomedicas (CRIB), Castilla La Mancha University, Albacete, Spain; ^2^ Yale Cancer Center, Yale University, New Haven, USA; ^3^ Division of Medical Oncology and Hematology, Princess Margaret Cancer Centre, University of Toronto, Toronto, Canada; ^4^ Semmelweis University, 2nd Department of Pediatrics, Budapest, Hungary; ^5^ MTA TTK Lendület Cancer Biomarker Research Group, Budapest, Hungary; ^6^ Cancer Research Center and CIBERONC, CSIC-University of Salamanca, Salamanca, Spain

**Keywords:** breast cancer, luminal A, mitotic kinases, clinical outcome

## Abstract

Luminal breast tumors have been classified into A and B subgroups, with the luminal A being associated with a more favorable clinical outcome. Unfortunately, luminal A tumors do not have a universally good prognosis. We used transcriptomic analyses using public datasets to evaluate the differential expression between normal breast tissue and breast cancer, focusing on upregulated genes included in cell cycle function. Association of selected genes with relapse free survival (RFS) and overall survival (OS) was performed using the KM Plotter Online Tool (http://www.kmplot.com). Seventy-seven genes were differentially expressed between normal and malignant breast tissue. Only five genes were associated with poor RFS and OS. The mitosis-related genes GTSE1, CDCA3, FAM83D and SMC4 were associated with poor outcome specifically in Luminal A tumors. The combination of FAM83D and CDCA3 for RFS and GTSE1 alone for OS showed the better prediction for clinical outcome. CDCA3 was amplified in 3.4% of the tumors, and FAM83D and SMC4 in 2.3% and 2.2%, respectively. In conclusion, we describe a set of genes that predict detrimental outcome in Luminal A tumors. These genes may have utility for stratification in trials of antimitotic agents or cytotoxic chemotherapy, or as candidates for direct target inhibition.

## INTRODUCTION

Breast cancer has been classified in different subtypes by gene expression analyses, and each of these subtypes is associated with different clinical outcomes [1, 2]. The luminal subtype includes those tumors that express the estrogen receptor, and therefore can be targeted with hormonal therapy [1, 3]. This subgroup has been classified as luminal A and B, and similarly these two subtypes are associated with different clinical outcomes [1, 4]. Luminal B tumors are linked with a more aggressive phenotype while luminal A shows a more benign behavior [4]. Luminal B tumors are characterized by an increased in proliferation, which is evidenced by elevated levels of Ki67 [5]. In addition, Luminal B tumors respond better to cytotoxic chemotherapy than the luminal A subtype.

It is known that the classification of breast cancer can stratify risk and prognosis; however not all tumors within a subgroup have similar clinical behavior. For instance, a subset of luminal A cancers are associated with poor outcome. Therefore the identification of these tumors could help to optimize therapy and to explore novel therapeutic strategies.

One of the main differences between the two luminal subgroups is the capacity of tumors to proliferate. However, measures of proliferation such as Ki67 are imperfect and some tumors that depend of mitosis are not identified. Therefore, the recognition of luminal A tumors that are mitotically more active and associated with worse outcome could help clinicians to customize their therapeutic decisions.

We hypothesize that by evaluating dysregulated genes involved in cell cycle and that are overexpressed in other breast cancer subtypes, we can identify a set of genes that would permit us to select those luminal A tumors associated with worse outcome. The identification of this subgroup would have important clinical implications as it could help determine which patients will respond poorly to endocrine therapy, opening the possibility to explore other therapeutic options in this group.

## RESULTS

### Transcriptomic analysis identify upregulated genes linked with cell cycle

**Figure 1:**
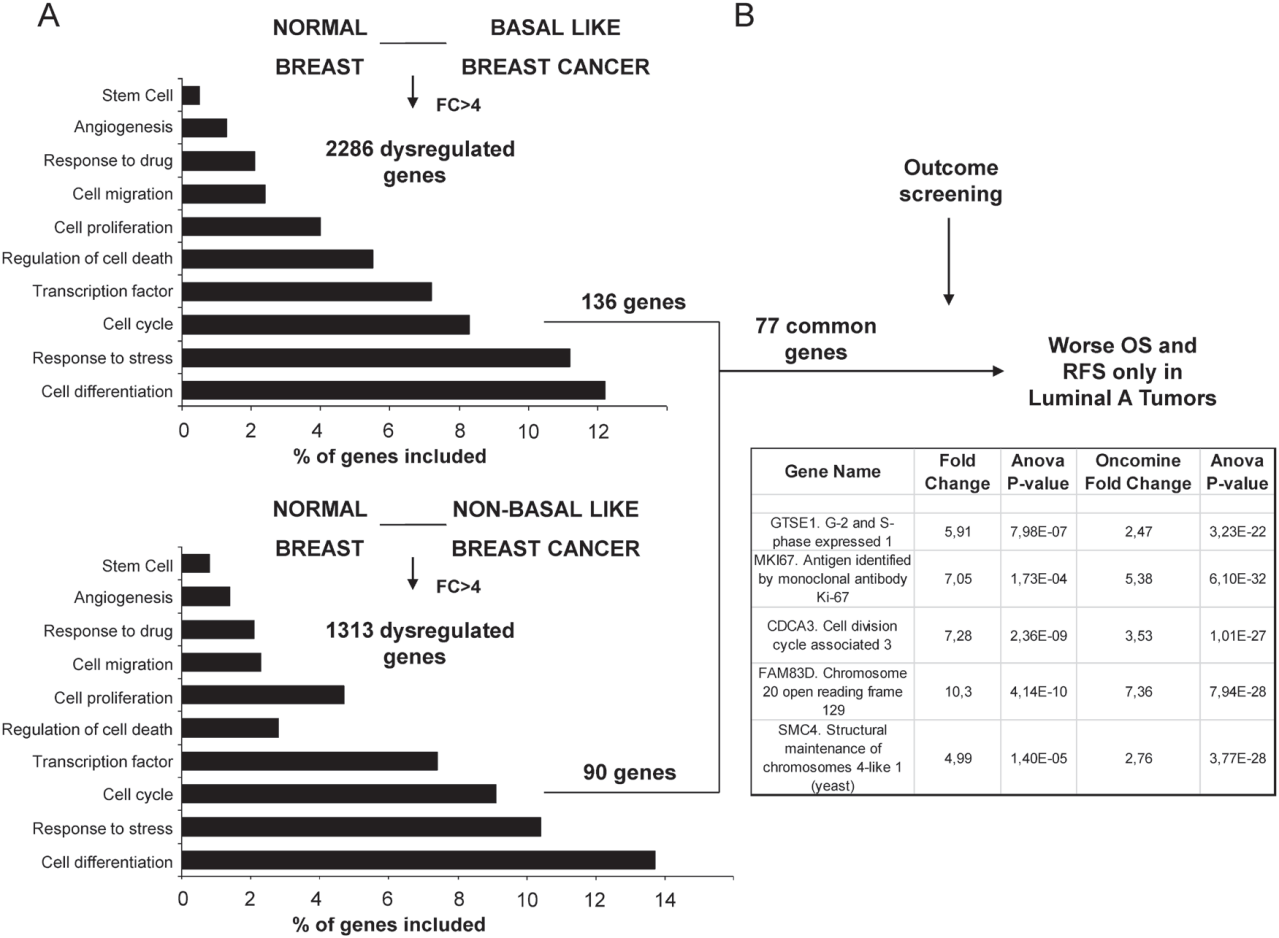
Identification of genes associated with detrimental outcome in Luminal A tumors **A**. Transcriptomic expression and pathway analyses among normal breast and basal-like and non-basal-like breast cancers, with the identification of dysregulated genes with more than ≥4 fold change included in the cell cycle gene ontology categories. **B**. Outcome screening for detrimental relapse free survival and overall survival. Confirmation of expression values between normal breast and breast cancer using data contained at Oncomine.

By comparing gene expression data from normal breast tissue and breast cancers, using a minimum fold change of 4, we identified 136 and 90 dysregulated genes included in the cell cycle function, in basal and non-basal breast cancers, respectively. Functional genomics using DAVID bioinformatic resources 6.7 identified several functions among the gene candidates (Figure 1A). A total number of 77 genes associated with cell cycle were shared among both basal and non-basal cancers (Figure 1A).

### Mitotic-related genes GTSE, CDCA3, FAM83D and SMC4 are associated with poor outcome

Using the online tool KM plotter (http://www.kmplot.com) [6] we selected genes that were associated with detrimental outcome specifically in luminal A tumors. The definition of breast cancer subtypes is described in material and methods. Among the 77 genes identified only 5 were associated with poor relapse free survival (RFS) and overall survival (OS) in the luminal A subtype (Figure 1B). One of the identified genes was MKi67, the gene that codes for Ki67. Therefore, we did not consider it for further outcome analyses as it is the gene used in the clinical setting to discriminate luminal A from luminal B subgroups. Of note, the predictive capacity for this gene was lower than the others selected, mainly for OS (HR: 1.4, CI: 1.17-1.67; log-rank p=0.00023 and HR: 1.47, CI: 1.01-2.16; log-rank p=0.046, for RFS and OS, respectively).

**Figure 2:**
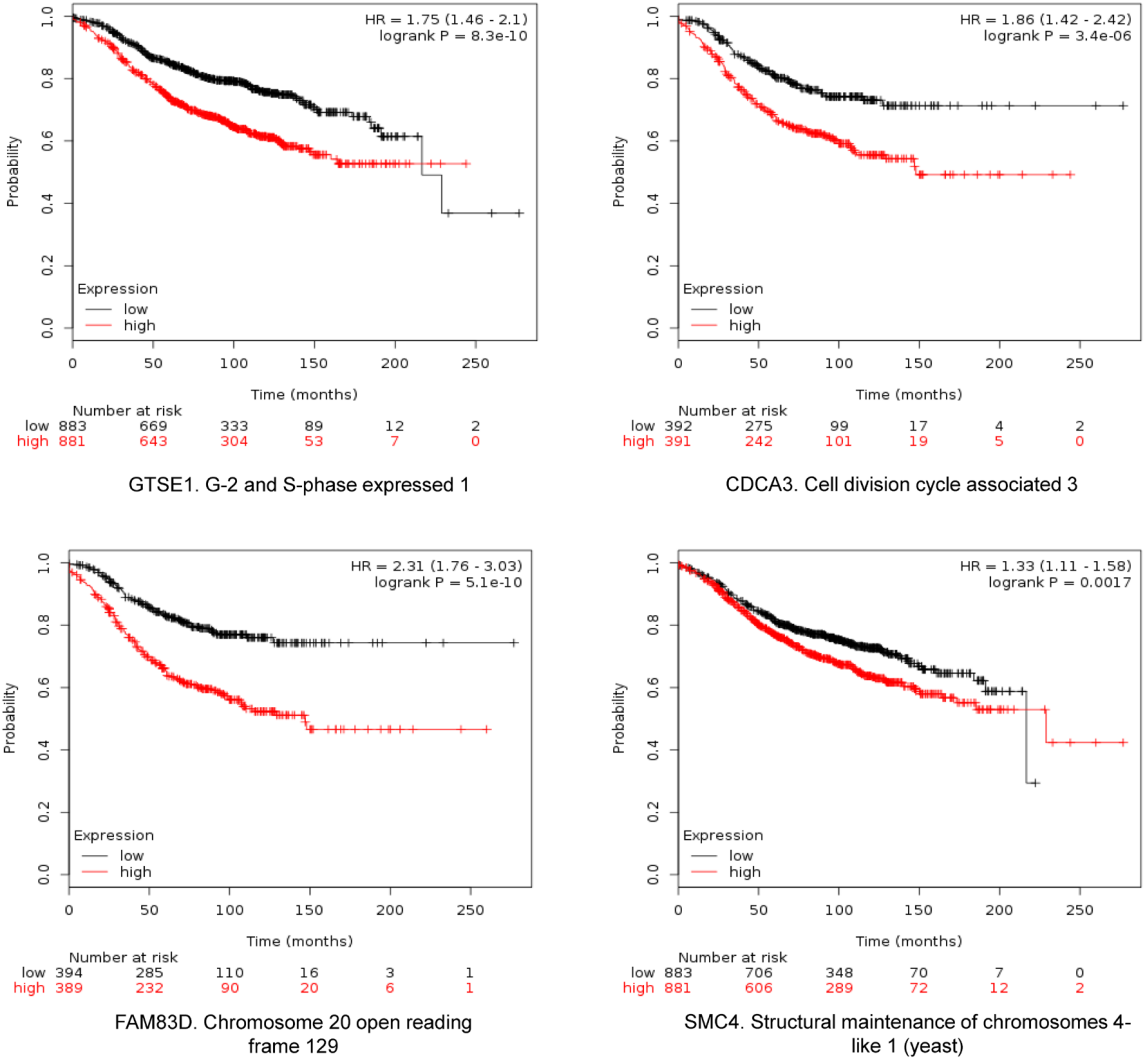
Association of GTSE, CDCA3, FAM83D and SMC4 individually with relapse free survival in Luminal A tumors using KM Plotter online tool, as described in material and methods

**Figure 3:**
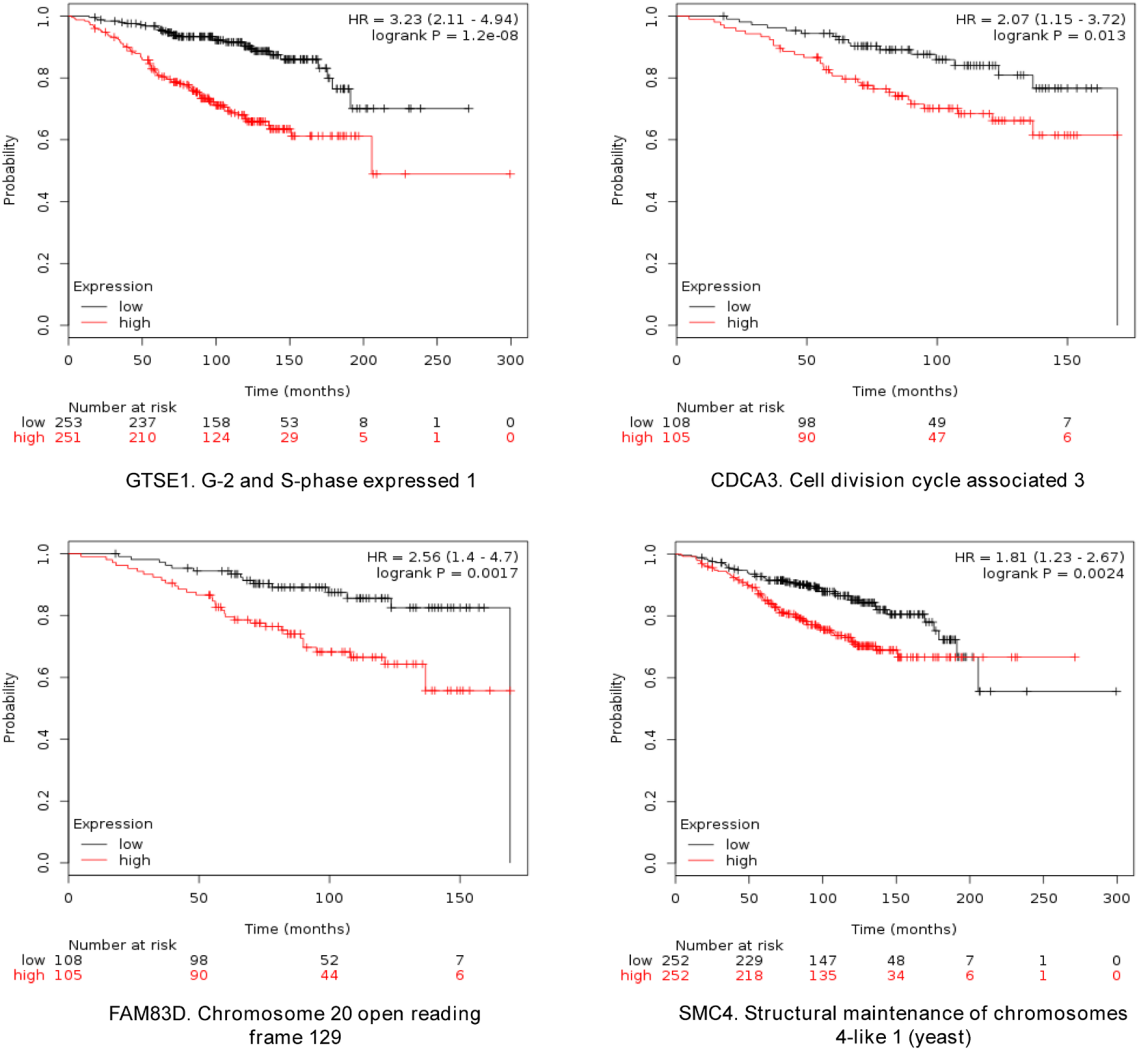
Association of GTSE, CDCA3, FAM83D and SMC4 individually with overall survival in Luminal A tumors, using KM Plotter online tool, as described in material and methods

The four selected genes were GTSE1, CDCA3, FAM83D and SMC4. Each gene was associated with detrimental RFS and OS in luminal A tumors as shown in Figure 2 and 3, respectively (additional data is shown in Supplementary Table 1 and 2, respectively). The upregulated expression of these genes in breast cancer was confirmed using data contained at Oncomine (Figure 1B).

### Combined analyses of GTSE1, CDCA3, FAM83D and SMC4 predicts poor RFS and OS in Luminal A tumors

**Figure 4:**
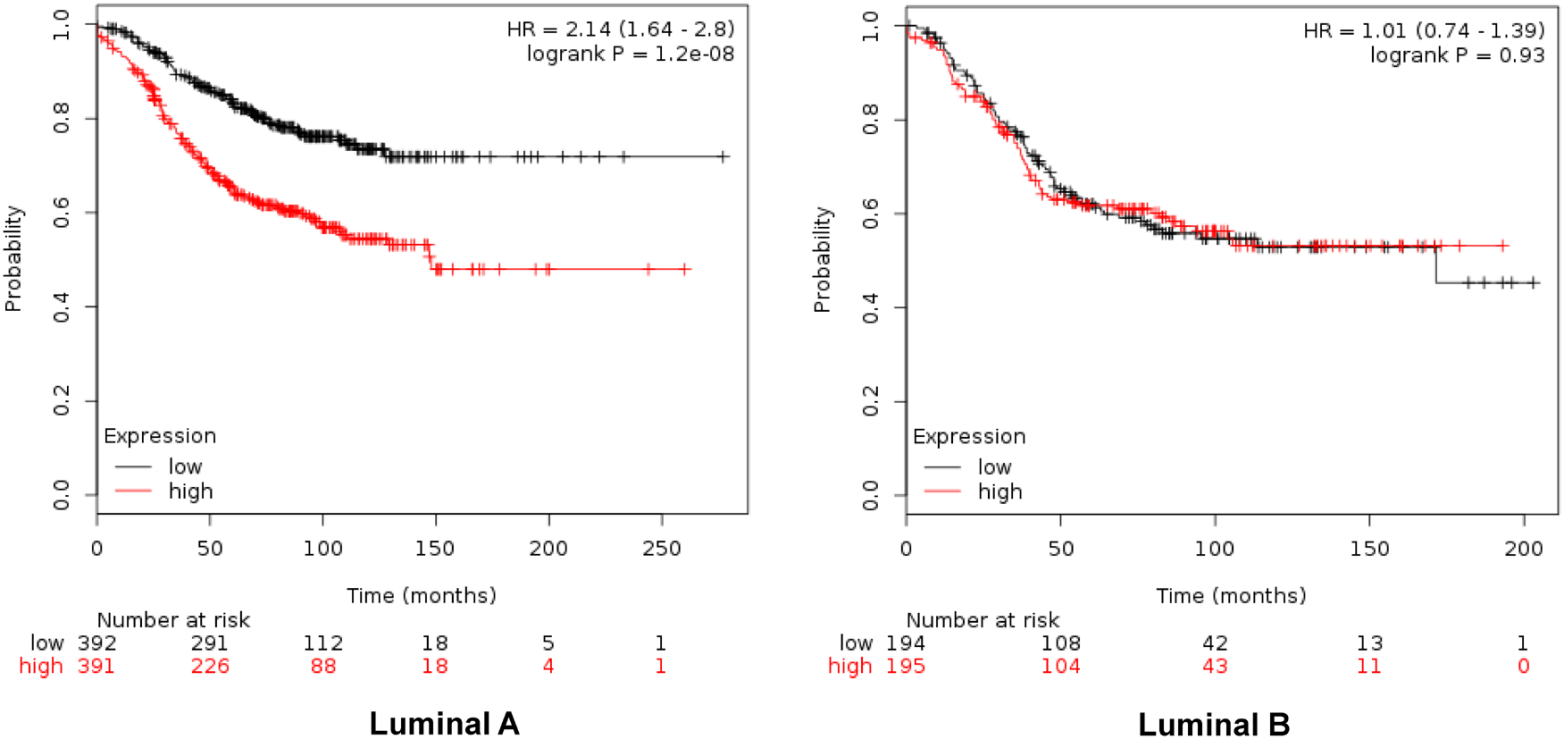
Association of the combined analyses of GTSE, CDCA3, FAM83D and SMC4 with relapse free survival in Luminal A. and B. tumors using KM Plotter online tool, as described in material and methods

**Figure 5:**
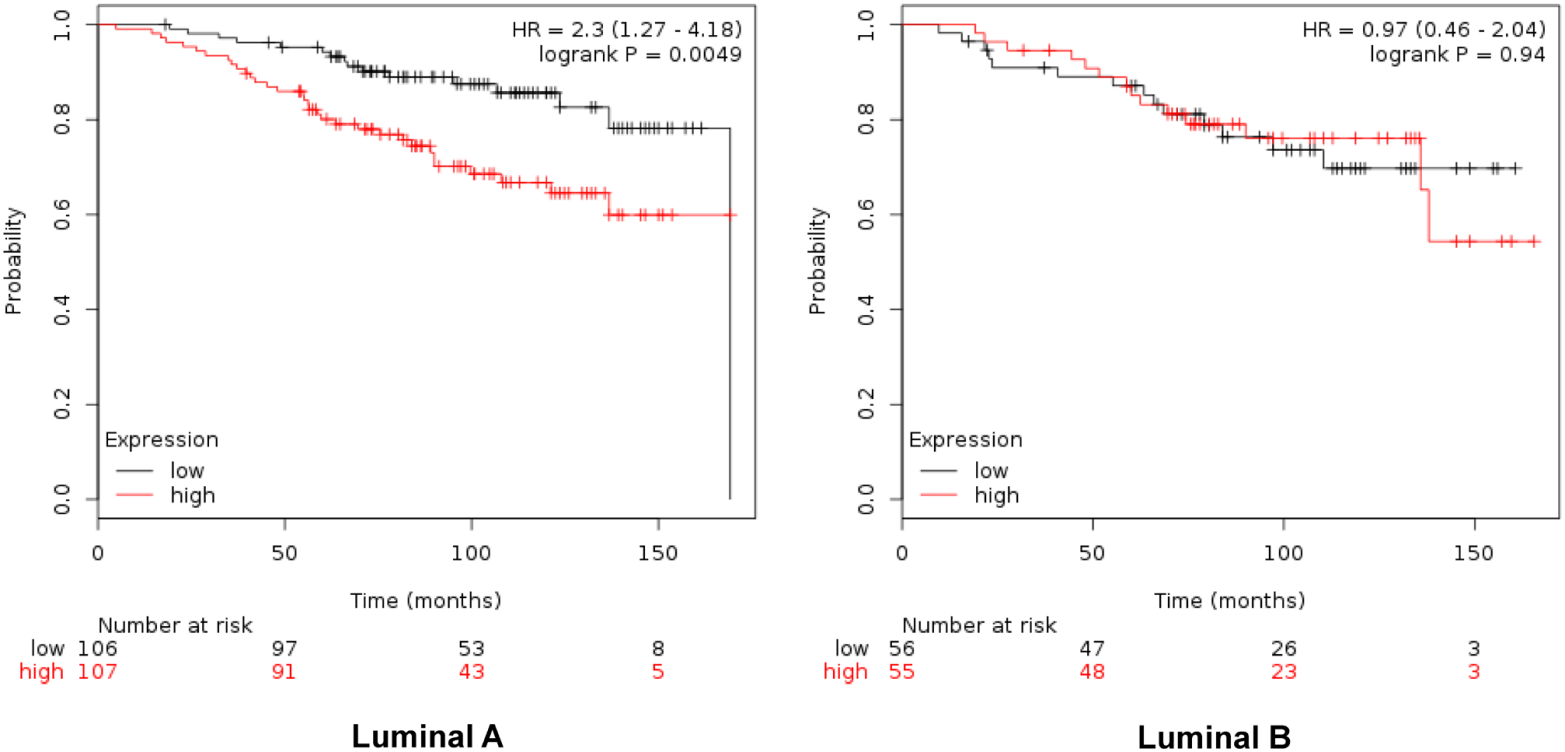
Association of the combined analyses of GTSE, CDCA3, FAM83D and SMC4 with overall survival in Luminal A. and B. tumors using KM Plotter online tool, as described in material and methods

When combined, the expression of GTSE1, CDCA3, FAM83D and SMC4 was significantly associated with worse RFS (HR 2.14, CI: 1.64-2.8; log rank p=1.2e-08) (Figure 4). Additionally, the combination of CDCA3 and FAM83D showed the highest effect on RFS (HR 2.34, CI: 1.78-3.08; log rank p=3.60e-10) (Table 1A). No association between these genes and RFS was observed for luminal B tumors (Supplementary Table 1). Similarly, a detrimental effect was observed for OS with the combined analyses of the four genes (HR 2.3; CI: 1.27-4.18; log rank p=0.0049) (Figure 5), but GTSE1 alone predicted the greatest magnitude of effect (HR 3.23; CI: 2.11-4.94; log rank p=1.2e-08) (Figure 3, and Supplementary Table 2). Of note, neither combination among genes was better than FAM83D and CDCA3 for RFS, and GTSE1 for OS (Table 1A and 1B, and Supplementary Table 2, respectively).

**Table 1:** A. Association with Relapse Free Survival of gene combinations in Luminal A tumors. B. Association with Overall Survival of gene combinations in Luminal A tumors

**A**		
**Relapse Free Survival. Luminal A**		
**Gene Symbols**	**Hazard Ratio**	**P-value**
GTSE1 + CDCA3	**1.82**	**7.40E-06**
GTSE1 + FAM83D	**2.09**	**3.80E-08**
GTSE1 + SMC4	**1.41**	**1.40E-04**
CDCA3 + FAM83D	**2.34**	**3.60E-10**
CDCA3 + SMC4	**1.7**	**6.20E-05**
FAM83D + SMC4	**1.85**	**3.40E-06**
CDCA3 + FAM83D + GTSE1	**2.03**	**1.40E-07**
CDCA3 + FAM83D + SMC4	**1.97**	**3.60E-07**
FAM83D + GTSE1 + SMC4	**1.91**	**9.90E-07**
**B**		
**Overall Survival. Luminal A**		
**Gene Symbols**	**Hazard Ratio**	**P-value**
GTSE1 + CDCA3	**2.02**	**0.017**
GTSE1 + FAM83D	**2.48**	**2.40E-03**
GTSE1 + SMC4	**2.13**	**1.30E-04**
CDCA3 + FAM83D	**2.22**	**7.20E-03**
CDCA3 + SMC4	**1.99**	**0.019**
FAM83D + SMC4	**2.17**	**9.10E-03**
CDCA3 + FAM83D + GTSE1	**2.57**	**1.60E-03**
CDCA3 + FAM83D + SMC4	**2.26**	**6.00E-03**
FAM83D + GTSE1 + SMC4	**2.52**	**2.00E-03**

KM Plotter online tool was used as described in material and methods

### Association with outcome by nodal status and treatment with chemotherapy

Next we evaluated the association of these genes alone or in combination with outcome by nodal status and chemotherapy treatment. GTSE1 and the combination of GTSE1, SMC4, FAM83D and CDCA3 predicted detrimental outcome for RFS in patients with both axillary positive and negative lymph nodes (Supplementary Table 3). Expression of GTSE1 was associated with poor OS in both subgroups. Similarly, GTSE1 predicted for detrimental RFS and OS in patients treated with or without chemotherapy, and the combination of the four genes predicted for RFS (Supplementary Table 3).

### Molecular alterations or copy number gains in the evaluated genes and potential druggable targets

Supplementary Table 4 describes the function of these genes. Finally, we evaluated if the association of these genes with outcome could be related to molecular alterations such as mutations or copy number modifications. CDCA3 was amplified in 3.4% of the tumors, and FAM83D and SMC4 in 2.3% and 2.2%, respectively (Supplementary Table 5). Finally using the Drug Gene Interaction Database (DGIdb) (http://dgidb.genome.wustl.edu/) we explored compounds that could potentially interact with the identified genes. Only the compound hydrochlorothiazide showed potential for interaction with CDCA3. No compound was found for potential interaction with GTSE1, FAM83D or SMC4.

## DISCUSSION

We have discovered a set of genes that identify luminal A patients with worse outcome. It is known that most patients that harbor luminal A tumors are treated with endocrine therapy. However, a subset of these tumors does not respond adequately to this treatment and have poor prognosis. In this context, the identification of a subgroup of patients within the luminal A subtype with a poor prognosis could help to stratify patients and consider alternative therapeutic strategies for them.

In our study by using transcriptomic analyses we identified genes that are overexpressed in breast cancer and linked with cell division. Among the genes commonly shared in the analyses, only four were associated with poor RFS and OS in luminal A tumors. Of note, the analyses of GTSE1 for OS and FAM83D and CDCA3 for RFS showed the worse outcome, an observation seen only in luminal A tumors.

CDCA3, FAM83D and SMC4 genes were found to be amplified in a very small percentage of tumors. The reduced presence of molecular alterations or copy modifications at a genomic level, lead us to consider these markers as an indirect measure of tumors with high proliferation capacity. It can be considered that these genes are not oncogenic drivers but may be indirect indicators of tumors that are more dependent of cell division and mitosis. In this context, tumors with these markers could be more sensitive to agents that target cell cycle or even cytotoxic chemotherapy. Therefore, these genes may have utility in identifying patients for such treatments.

CDCA3 has been associated with both cancer risk [7] and poor prognosis in certain tumors [8]. High levels of FAM83D have been associated with poor outcome in several tumors and an increase in proliferation *in vitro* models [9, 10]. SMC4 belongs to a family of genes linked with poor outcome in prostate cancer [11], and finally, GTSE1 was described as associated with worse prognosis in uterine leimyosarcoma [12].

It should be mentioned that one of the identified genes that was differentially expressed was MKi67, the gene that encodes for Ki67. As this gene is currently used to select between luminal A and B tumors, therefore it was not included in our evaluation. However, the magnitude of association between the expression of MKi67 and poor outcome was lower than the selected genes.

In addition it should be noted that the genes identified are not druggable targets as can be seen from our analyses, with the exception of hydrochlorothiazide that could have a potential interaction with CDCA3.

A limitation of our study is a potential heterogeneity in the treatment the patients received. Although it can be expected that most estrogen-positive patients were treated with endocrine therapy, this cannot be confirmed. Also, the results are based on univariable analysis as there was insufficient information on patient and tumor characteristics to inform a multivariable model. This, in addition to survival data which were available for only a small number of patients, suggests that results should be considered hypotheses generating and should be validated in a large independent cohort. Our analysis focused on proliferation. It is known that other biological functions beyond mitosis can differentiate between luminal A and B tumors. Finally, it should be mentioned that information contained at Oncomine does not distinguish among breast cancer subtypes.

In conclusion, we describe a set of genes that are overexpressed in luminal A tumors that predict for detrimental outcome. These biomarkers could help to stratify therapies including treatment with antimitotic agents or cytotoxic chemotherapy.

## MATERIALS AND METHODS

### Transcriptomic analysis and identification of upregulated genes

mRNA level data from normal breast tissue and basal-like and non-basal like breast tumors were extracted from a public dataset (GEO DataSet accession number: GDS2250)[13, 14]. Affymetrix CEL files were downloaded and analyzed with Affymetrix Transcriptome Analysis Console 3.0. Only genes with minimum 4-fold differential expression values between control and other groups were selected. The list of genes was analyzed using gene set enrichment analyses using DAVID Bioinformatics Resources 6.7 in order to identify functions of these genes (https://david.ncifcrf.gov/). We used an adjusted p-value <0.05 to select the enriched gene-sets. The differentially expressed genes were independently confirmed using data contained at Oncomine (www.oncomine.org) (TCGA Breast). For this analysis, due to the absence of breast cancer subtypes in this dataset we compared normal breast with breast cancer.

### Outcome analyses

The KM Plotter Online Tool (http://www.kmplot.com)[6] was used to evaluate the relationship between the presence of different genes and patient clinical outcome in different breast cancer subtypes.

This publicly available database allows to investigate overall survival (OS) and relapse-free survival (RFS) in luminal A, luminal B and basal-like breast cancers.

### Definitions of breast cancer subtypes

Breast cancer subtypes in the KM Plotter Online tool are defined as follow: Triple negative: ESR1-/HER2-. Luminal A: ESR1+/HER2-/MKI67 low. Luminal B: ESR1+/HER2-/MKI67 high and ESR1+/HER2+ -.

### Evaluation of molecular alterations

We used data contained at cBioportal (www.cbioportal.org) (TCGA dataset) to explore the role of mutations, deletions or amplifications in the identified genes [15].

### Evaluation of gene-drug interactions

For the evaluation of compounds that could potentially interact with the identified genes we used the Drug Gene Interaction Database (DGIdb) (http://dgidb.genome.wustl.edu/).

## SUPPLEMENTARY MATERIALS FIGURES AND TABLES








