# Supplementary material for: Mitotic read-out genes confer poor outcome in luminal A breast cancer tumors
Source: Oncotarget. 2017 Feb 21;8(13):21733–40. doi: 10.18632/oncotarget.15562 (PMC5400619; doi:10.18632/oncotarget.15562)
Supplement: Supplementary file 1 [file oncotarget-08-21733-s001.pdf]

Mitotic read-out genes confer poor outcome in luminal A breast cancer tumors

SUPPLEMENTARY TABLES

Supplementary Table 1: List of upregulated genes and association with outcome in different breast cancer subtypes. KM Plotter online tool was used as described in material and methods

See Supplementary File 1

Supplementary Table 2: List of upregulated genes and association with outcome in different breast cancer subtypes. KM Plotter online tool was used as described in material and methods

See Supplementary File 2

Supplementary Table 3: Association with Relapse Free Survival and Overall Survival of GTSE, CDCA3, FAM83D and SMC4 individually or in combination, in Luminal A tumors by nodal status and chemotherapy treatment. KM Plotter online tool was used as described in material and methods

See Supplementary File 3

Supplementary Table 4: Biological functions of the identified genes

Probe_Set	Gene name	Gene symbol	Function
204318_s_at	GTSE1. G-2 and S-phase expressed 1.	GTSE1, GTSE-1, B99	May be involved in p53-induced cell cycle arrest in G2/M phase by interfering with microtubule rearrangements that are required to enter mitosis. Overexpression delays G2/M phase progression.
223307_at	CDCA3. Cell division cycle associated 3.	CDCA3, C8, Grcc8, Tome1	F-box-like protein which is required for entry into mitosis. Acts by participating in E3 ligase complexes that mediate the ubiquitination and degradation of WEE1 kinase at G2/M phase.
225687_at	FAM83D. Chromosome 20 open reading frame 129.	FAM83D, C20orf129	Probable proto-oncogene that regulates cell proliferation, growth, migration and epithelial to mesenchymal transition. Through the degradation of FBXW7, may act indirectly on the expression and downstream signaling of MTOR, JUN and MYC (PubMed:24344117). May play also a role in cell proliferation through activation of the ERK1/ERK2 signaling cascade (PubMed:25646692). May also be important for proper chromosome congression and alignment during mitosis through its interaction with KIF22.
201663_s_at	SMC4. Structural maintenance of chromosomes 4-like 1 (yeast).	SMC4, SMCAL1, CAP-C, hCAP-C	Central component of the condensin complex, a complex required for conversion of interphase chromatin into mitotic-like condense chromosomes. The condensin complex probably introduces positive supercoils into relaxed DNA in the presence of type I topoisomerases and converts nicked DNA into positive knotted forms in the presence of type II topoisomerases.

Supplementary Table 5: Molecular alterations including copy number gains and mutations in the identified genes

974 Breast Invasive Carcinoma Samples			
**Gene Name**	**Amplification**	**Deletion**	**Mutation**
GTSE1. G-2 and S-phase expressed 1.	0.8%	0.6%	0.4%
MKI67. Antigen identified by monoclonal antibody Ki-67.	0.6%	0.8%	1.4%
CDCA3. Cell division cycle associated 3.	3.4%	0.2%	0.1%
FAM83D. Chromosome 20 open reading frame 129.	2.3%	-	-
SMC4. Structural maintenance of chromosomes 4-like 1 (yeast).	2.2%	-	0.8%

Data contained at cBioportal (www.cbioportal.org) was used for the analyses.
