# Supplementary material for: Mitotic read-out genes confer poor outcome in luminal A breast cancer tumors
Source: Oncotarget. 2017 Feb 21;8(13):21733–40. doi: 10.18632/oncotarget.15562 (PMC5400619; doi:10.18632/oncotarget.15562)
Supplement: Supplementary file 2 [file oncotarget-08-21733-s002.doc]

| **Probe Set** | **Gene Name** | **Relapse Free Survival** | | | | | | | |
| --- | --- | --- | --- | --- | --- | --- | --- | --- | --- |
|  |  | **Basal Like** | | **Luminal A** | | **Luminal B** | | **HER2+** | |
|  |  | **HR (Hazard Ratio)** | **Logrank P value** | **HR (Hazard Ratio)** | **Logrank P value** | **HR (Hazard Ratio)** | **Logrank P value** | **HR (Hazard Ratio)** | **Logrank P value** |
| **204170_s_at** | [**CDC28 protein kinase regulatory subunit 2**](https://david.ncifcrf.gov/geneReportFull.jsp?rowids=817048) | **1,06 (0,82 - 1,38)** | **0.64** | **1,83 (1,53 - 2,2)** | **3.20E-11** | **1,43 (1,17-1,76)** | **0.00049** | **1,18 (0,78-1,79)** | **0.43** |
| **204126_s_at** | [**CDC45 cell division cycle 45-like (S. cerevisiae)**](https://david.ncifcrf.gov/geneReportFull.jsp?rowids=813733) | **1,02 (0,79-1,33)** | **0.86** | **1,79 (1,49 -2,14)** | **1.60E-10** | **1,46 (1,19-1,79)** | **0.00023** | **1,24 (0,82-1,89)** | **0.3** |
| **222179_at** | [**CDC5 cell division cycle 5-like (S. pombe)**](https://david.ncifcrf.gov/geneReportFull.jsp?rowids=821458) | **0,72 (0,55 - 0,94)** | **0.014** | **0,74 (0,62-0,88)** | **0.00076** | **0,8 (0,65-0,97)** | **0.027** | **0,91 (0,6-1,37)** | **0.64** |
| **213008_at** | [**Fanconi anemia, complementation group I**](https://david.ncifcrf.gov/geneReportFull.jsp?rowids=823221) | **1,13 (0,87-1,47)** | **0.36** | **1,96 (1,63-2,35)** | **2.30E-13** | **1,51 (1,23-1,85)** | **5.80E-05** | **1,17 (0,77-1,78)** | **0.45** |
| **204318_s_at** | **GTSE1. G-2 and S-phase expressed 1** | **0,83 (0,64-1,08)** | **0.16** | **1,75 (1,46-2,1)** | **8.30E-10** | **1,06 (0,87-1,3)** | **0.57** | **0,85 (0,56-1,28)** | **0.43** |
| **213524_s_at** | [**G0/G1switch 2**](https://david.ncifcrf.gov/geneReportFull.jsp?rowids=777021) | **1 (0,77-1,3)** | **0.99** | **0,85 (0,71-1,01)** | **0.067** | **0,86 (0,7-1,05)** | **0.13** | **0,85 (0,56-1,29)** | **0.44** |
| **218726_at** | [**Holliday junction recognition protein**](https://david.ncifcrf.gov/geneReportFull.jsp?rowids=824595) | **0,94 (0,73-1,22)** | **0.66** | **1,99 (1,66-2,4)** | **8.40E-14** | **1,24 (1,01-1,52)** | **0.036** | **1,05 (0,69-1,59)** | **0.82** |
| **206205_at** | [**M-phase phosphoprotein 9**](https://david.ncifcrf.gov/geneReportFull.jsp?rowids=777293) | **0,85 (0,65-1,1)** | **0.21** | **1,28 (1,07-1,53)** | **0.0058** | **1,38 (1,13-1,69)** | **0.0016** | **0,92 (0,61-1,4)** | **0.7** |
| **203362_s_at** | **MAD2L1. MAD2 mitotic arrest deficient-like 1 (yeast)** | **1.27 (0.98 − 1.65)** | **0.068** | **2.06 (1.72 − 2.48)** | **2.6E−15** | **1.52 (1.24 − 1.86)** | **5.3e−05** | **1.08 (0.71 − 1.64)** | **0.71** |
| **204162_at** | **KNTC2. Kinetochore associated 2** | **1.27 (0.98 − 1.65)** | **0.071** | **2.11 (1.76 − 2.54)** | **3.3E−16** | **1.43 (1.17 − 1.76)** | **4.50E-04** | **0.75 (0.49 − 1.14)** | **0.17** |
| **204641_at** | [**NIMA (never in mitosis gene a)-related kinase 2**](https://david.ncifcrf.gov/geneReportFull.jsp?rowids=791562) | **1,24 (0,96 - 1,61)** | **0.1** | **1,99 (1,66 - 2,4)** | **4.90E-14** | **1,58 (1,29 - 1,94)** | **8.80E-06** | **1,47 (0,97 - 2,24)** | **0.07** |
| **223381_at** | [**NUF2, NDC80 kinetochore complex component, homolog (S. cerevisiae)**](https://david.ncifcrf.gov/geneReportFull.jsp?rowids=809559) | **1,13 (0,81-1,59)** | **0.46** | **1,99 (1,53-2,6)** | **2.10E-07** | **1,24 (0,91-2,7)** | **0.18** | **1,01 (0,63-1,63)** | **0.95** |
| **219148_at** | [**PDZ binding kinase**](https://david.ncifcrf.gov/geneReportFull.jsp?rowids=816657) | **0,96 (0,74-1,24)** | **0.75** | **1,91 (1,59-2,29)** | **1.50E-12** | **1,49 (1,22-1,83)** | **1.00E-04** | **0,72 (0,47-1,09)** | **0.12** |
| **204558_at** | [**RAD54-like (S. cerevisiae)**](https://david.ncifcrf.gov/geneReportFull.jsp?rowids=799631) | **1,12 (0,86-1,45)** | **0.4** | **1,54 (1,29-1,85)** | **1.50E-06** | **1,34 (1,09-1,64)** | **0.0045** | **0,98 (0,65-1,49)** | **0.94** |
| **222077_s_at** | [**Rac GTPase activating protein 1 pseudogene; Rac GTPase activating protein 1**](https://david.ncifcrf.gov/geneReportFull.jsp?rowids=795700) | **1,53 (1,18-1,99)** | **1.30E-03** | **2,08 (1,73-2,49)** | **1.10E-15** | **1,74 (1,42-2,14)** | **7.70E-08** | **1,33 (0,88-2,02)** | **0.18** |
| **235572_at** | [**SPC24, NDC80 kinetochore complex component, homolog (S. cerevisiae)**](https://david.ncifcrf.gov/geneReportFull.jsp?rowids=803549) | **0,88 (0,63-1,23)** | **0.46** | **1,65 (1,27-2,15)** | **0.00015** | **1,41 (1,03-1,93)** | **0.033** | **0,79 (0,49-1,27)** | **0.33** |
| **209891_at** | [**SPC25, NDC80 kinetochore complex component, homolog (S. cerevisiae)**](https://david.ncifcrf.gov/geneReportFull.jsp?rowids=821580) | **1,08 (0,83-1,4)** | **0.56** | **1,6 (1,34-1,92)** | **2.00E-07** | **1,26 (1,03-1,54)** | **0.025** | **0,97 (0,64-1,47)** | **0.87** |
| **210052_s_at** | **TPX2. Microtubule-associated protein homolog (Xenopus laevis)** | **0.89 (0.69 − 1.16)** | **0.39** | **1.94 (1.62 − 2.33)** | **4.9E−13** | **1.33 (1.09 − 1.63)** | **5.20E-03** | **0.76 (0.5 − 1.15)** | **0.19** |
| **204822_at** | [**TTK protein kinase**](https://david.ncifcrf.gov/geneReportFull.jsp?rowids=814656) | **1,09 (0,84 - 1,42)** | **0.51** | **2,03 (1,69 - 2,43)** | **9.0E-15** | **1,42 (1,16 - 1,74)** | **6.00E-04** | **0,95 (0,63 - 1,45)** | **0.82** |
| **204026_s_at** | [**ZW10 interactor**](https://david.ncifcrf.gov/geneReportFull.jsp?rowids=783593) | **0,98 (0,75 - 1,27)** | **0.87** | **1,78 (1,49 - 2,13)** | **2.4E-10** | **1,54(1,25 - 1,88)** | **2.9E-05** | **1,03 (0,68 - 1,56)** | **0.9** |
| **222608_s_at** | **ANLN. Anillin, actin binding protein (scraps homolog, Drosophila)** | **1.41 (1 − 1.97)** | **0.048** | **2.16 (1.65 − 2.82)** | **1.1E−08** | **1.33 (0.97 − 1.82)** | **0.074** | **1.41 (0.88 − 2.27)** | **0.15** |
| **212023_s_at** | **MKI67. Antigen identified by monoclonal antibody Ki-67** | **0,81 (0,62 - 1,05)** | **0.11** | **1,4 (1,17 - 1,67)** | **2.30E-04** | **1,19 (0,97 - 1,45)** | **0.095** | **0,85 (0,56 - 1,3)** | **0.46** |
| **219918_s_at** | **ASPM. Asp (abnormal spindle)-like, microcephaly associated (Drosophila)** | **1.26 (0.97 − 1.63)** | **0.085** | **2.02 (1.68 − 2.42)** | **1.6E−14** | **1.6 (1.31 − 1.96)** | **4.8e−06** | **0.95 (0.62 − 1.44)** | **0.8** |
| **204092_s_at** | [**AURKA. Aurora kinase A; aurora kinase A pseudogene 1**](https://david.ncifcrf.gov/geneReportFull.jsp?rowids=782105) | **0,95 (0,73 - 1,23)** | **0.69** | **1,99 (1,66 - 2,39)** | **6.00E-14** | **1,39 (1,13 - 1,7)** | **1.5E-03** | **0,7 (0,46 - 1,07)** | **0.1** |
| **210334_x_at** | **BIRC5. Baculoviral IAP repeat-containing 5 (survivin)** | **0.83 (0.64 − 1.08)** | **0.17** | **1.14 (0.95 − 1.36)** | **0.16** | **0.93 (0.76 − 1.13)** | **0.46** | **0.82 (0.54 − 1.24)** | **0.35** |
| **209642_at** | **BUB1. Budding uninhibited by benzimidazoles 1 homolog (yeast)** | **1 (0,77 - 1,29)** | **0.98** | **2,2 (1,83 - 2,65)** | **<1.0E-16** | **1,41 ( 1,15 - 1,73)** | **8.7E-04** | **0.8 (0.53 - 1.21)** | **0.29** |
| **203755_at** | [**budding uninhibited by benzimidazoles 1 homolog beta (yeast)**](https://david.ncifcrf.gov/geneReportFull.jsp?rowids=775002) | **1.1 (0.85 - 1.43)** | **0.46** | **2,27 (1,89 - 2,72)** | **<1E-16** | **1,47 (1,2 - 1,8)** | **2.00E-04** | **1,01 (0,67 - 1,54)** | **0.94** |
| **210559_s_at** | [**cell division cycle 2, G1 to S and G2 to M**](https://david.ncifcrf.gov/geneReportFull.jsp?rowids=815142) | **0,89 (0,69 - 1,16)** | **0.38** | **1,68 (1,41 - 2,02)** | **1.0E-08** | **1,43 (1,17 - 1,76)** | **4.7E-04** | **0,72 (0,47 - 1,09)** | **0.12** |
| **202870_s_at** | **CDC20. Cell division cycle 20 homolog (S. cerevisiae)** | **1.01 (0.78 − 1.31)** | **0.93** | **2.06 (1.71 − 2.48)** | **4.7E−15** | **1.47 (1.2 − 1.8)** | **1.90E-04** | **0.97 (0.64 − 1.46)** | **0.87** |
| **217010_s_at** | [**cell division cycle 25 homolog C (S. pombe)**](https://david.ncifcrf.gov/geneReportFull.jsp?rowids=817790) | **1,02 (0,79 - 1,32)** | **0.88** | **1,01 (0,84 - 1,2)** | **0.95** | **1 (0,82 - 1,23)** | **0.99** | **0,51 (0,33 - 0,78)** | **1.60E-03** |
| **223307_at** | **CDCA3. Cell division cycle associated 3** | **0.92 (0.65 − 1.29)** | **0.61** | **1.86 (1.42 − 2.42)** | **3.4E−06** | **1.25 (0.92 − 1.72)** | **0.16** | **0.84 (0.53 − 1.36)** | **0.48** |
| **224753_at** | [**cell division cycle associated 5**](https://david.ncifcrf.gov/geneReportFull.jsp?rowids=789361) | **1,04 (0,74 - 1,46)** | **0.82** | **2,36 (1,79 - 3,1)** | **2.2E-10** | **1,59 (1,16 - 2,19)** | **3.80E-03** | **0.88 (0.54 - 1.41)** | **0.58** |
| **204962_s_at** | **CENPA. Centromere protein A, 17kDa** | **1.07 (0.82 − 1.39)** | **0.61** | **2.24 (1.86 − 2.69)** | **< 1E−16** | **1.76 (1.43 − 2.16)** | **5.1e−08** | **0.74 (0.49 − 1.13)** | **0.16** |
| **205046_at** | [**centromere protein E, 312kDa**](https://david.ncifcrf.gov/geneReportFull.jsp?rowids=789965) | **1,33 (1,02 - 1,72)** | **0.032** | **1,98 (1,65 - 2,37)** | **7.0E-14** | **1,36 (1,11 - 1,66)** | **3.10E-03** | **1.13 (0.74 - 1.71)** | **0.57** |
| **207828_s_at** | **Centromere protein F, 350/400ka (mitosinCENPF. )** | **1.21 (0.93 − 1.57)** | **0.15** | **1.83 (1.53 − 2.19)** | **3.6E−11** | **1.52 (1.24 − 1.86)** | **5.2e−05** | **1.05 (0.7 − 1.6)** | **0.81** |
| **218542_at** | **CEP55. Chromosome 10 open reading frame 3** | **1 (0.77 − 1.3)** | **1** | **2.2 (1.83 − 2.65)** | **< 1E−16** | **1.53 (1.25 − 1.88)** | **3.5e−05** | **0.89 (0.59 − 1.36)** | **0.6** |
| **228868_x_at** | [**chromatin licensing and DNA replication factor 1**](https://david.ncifcrf.gov/geneReportFull.jsp?rowids=780175) | **1,02 (0,73 - 1,43)** | **0.92** | **1,76 (1,35 - 2,29)** | **1.9E-05** | **1,42 (1,04 - 1,95)** | **2.8E-02** | **0,75 (0,47 - 1,21)** | **0.24** |
| **227165_at** | [**chromosome 13 open reading frame 3**](https://david.ncifcrf.gov/geneReportFull.jsp?rowids=809850) | **1,04 (0,74 - 1,46)** | **0.83** | **1,54 (1,19 - 2)** | **1.1E-03** | **1,33 (0,97 - 1,83)** | **7.2E-02** | **0,78 (0,48 - 1,25)** | **0.3** |
| **244173_at** | [**chromosome 14 open reading frame 106**](https://david.ncifcrf.gov/geneReportFull.jsp?rowids=793643) | **0,92 (0,66 - 1,29)** | **0.63** | **0,91 (0,7 - 1,17)** | **0.45** | **1,04 (0,76 - 1,42)** | **0.82** | **0,86 (0,54 - 1,39)** | **0.55** |
| **217640_x_at** | [**chromosome 18 open reading frame 24**](https://david.ncifcrf.gov/geneReportFull.jsp?rowids=773124) | **1,05 (0,81 - 1,37)** | **0.7** | **1,42 (1,19 - 1,69)** | **1.1E-04** | **1,07 (0,87 - 1,3)** | **0.53** | **1,25 (0,83 - 1,9)** | **0.29** |
| **203418_at** | **CCNA2. Cyclin A2** | **0.91 (0.7 − 1.18)** | **0.46** | **1.49 (1.25 − 1.78)** | **1.1E−05** | **1.16 (0.95 − 1.42)** | **0.15** | **0.8 (0.53 − 1.22)** | **0.3** |
| **214710_s_at** | **CCNB1. Cyclin B1** | **1.37 (1.05 − 1.77)** | **0.019** | **1.72 (1.44 − 2.06)** | **2.6E−09** | **1.86 (1.51 − 2.29)** | **2.1e−09** | **1.3 (0.85 − 1.97)** | **0.22** |
| **202705_at** | [**cyclin B2**](https://david.ncifcrf.gov/geneReportFull.jsp?rowids=813528) | **1,22 (0,94 - 1,58)** | **0.14** | **2,33 (1,93 - 2,8)** | **<1E-16** | **1,64 (1,34 - 2,02)** | **1.4E-06** | **1,14 (0,75 - 1,73)** | **0.53** |
| **205034_at** | **CCNE2 Cyclin E2** | **1.19 (0.92 − 1.55)** | **0.19** | **2.12 (1.76 − 2.55)** | **2.2E−16** | **1.44 (1.18 − 1.77)** | **3.70E-04** | **1.45 (0.96 − 2.21)** | **0.078** |
| **209714_s_at** | [**cyclin-dependent kinase inhibitor 3**](https://david.ncifcrf.gov/geneReportFull.jsp?rowids=815446) | **1,13 (0,87 - 1,47)** | **0.34** | **1,96 (1,32 - 2,93)** | **7.10E-04** | **1,41 (1,15 - 1,73)** | **8.30E-04** | **1,01 (0,67 - 1,53)** | **0.96** |
| **238048_at** | [**cytoplasmic linker associated protein 2**](https://david.ncifcrf.gov/geneReportFull.jsp?rowids=794156) | **0,72 (0,51 - 1,01)** | **0.057** | **0,61 (0,47 - 0,79)** | **2.10E-04** | **0,6 (0,44 - 0,83)** | **1.70E-03** | **0,75 (0,47 - 1,2)** | **0.23** |
| **203764_at** | **DLGAP5. Discs, large homolog 7 (Drosophila)** | **1.22 (0.94 − 1.59)** | **0.13** | **2.32 (1.93 − 2.8)** | **< 1E−16** | **1.4 (1.15 − 1.72)** | **1.00E-03** | **0.83 (0.55 − 1.26)** | **0.38** |
| **204455_at** | **DST. Dystonin** | **1,04 (0,8 - 1,35)** | **0.77** | **0,7 (0,59 - 0,84)** | **1.00E-04** | **0,79 (0,64 - 0,96)** | **0.016** | **1,27 (0,84 - 1,93)** | **0.26** |
| **201983_s_at** | [**epidermal growth factor receptor (erythroblastic leukemia viral (v-erb-b) oncogene homolog, avian)**](https://david.ncifcrf.gov/geneReportFull.jsp?rowids=816368) | **1,25 (0,96 - 1,63)** | **0.09** | **0,94 (0,79 - 1,12)** | **0.5** | **0,93 (0,76 - 1,14)** | **0.49** | **0,93 (0,62 - 1,42)** | **0.75** |
| **219650_at** | [**excision repair cross-complementing rodent repair deficiency, complementation group 6-like**](https://david.ncifcrf.gov/geneReportFull.jsp?rowids=820324) | **1,11 (0,85 - 1,44)** | **0.44** | **1,73 (1,44 - 2,07)** | **1.70E-09** | **1,61 (1,31 - 1,98)** | **3.70E-06** | **0,95 (0,62 - 1,43)** | **0.8** |
| **204603_at** | [**exonuclease 1**](https://david.ncifcrf.gov/geneReportFull.jsp?rowids=776417) | **0,92 (0,71 - 1,2)** | **0.55** | **1,39 (1,16 - 1,66)** | **2.60E-04** | **1,45 (1,18 - 1,78)** | **3.10E-04** | **0,92 (0,61 - 1,4)** | **0.7** |
| **204817_at** | [**extra spindle pole bodies homolog 1 (S. cerevisiae)**](https://david.ncifcrf.gov/geneReportFull.jsp?rowids=802581) | **0,67 (0,52 - 0,88)** | **0.0031** | **1,69 (1,41 - 2,03)** | **9.60E-09** | **1,18 (0,97 - 1,45)** | **0.1** | **0,82 (0,54 - 1,24)** | **0.34** |
| **225687_at** | **FAM83D. Chromosome 20 open reading frame 129** | **1 (0.72 − 1.41)** | **0.98** | **2.31 (1.76 − 3.03)** | **5.1E−10** | **1.18 (0.86 − 1.61)** | **0.3** | **0.63 (0.39 − 1.02)** | **0.058** |
| **202580_x_at** | **FOXM1. Forkhead box M1** | **0.81 (0.63 − 1.06)** | **0.12** | **1.88 (1.56 − 2.25)** | **7.9E−12** | **1.51 (1.23 − 1.85)** | **6.7e−05** | **0.61 (0.4 − 0.94)** | **0.023** |
| **204456_s_at** | [**growth arrest-specific 1**](https://david.ncifcrf.gov/geneReportFull.jsp?rowids=794296) | **0,9 (0,69 - 1,17)** | **0.42** | **0,82 (0,68 - 0,97)** | **0.025** | **0,9 (0,73 - 1,1)** | **0.3** | **0,69 (0,45 - 1,06)** | **0.09** |
| **235709_at** | [**growth arrest-specific 2 like 3**](https://david.ncifcrf.gov/geneReportFull.jsp?rowids=787351) | **1,47 (1,05 - 2,07)** | **0.025** | **1,82 (1,4 - 2,37)** | **6.20E-06** | **2,25 (1,62 - 3,13)** | **6.30E-07** | **1,2 (0,75 - 1,93)** | **0.45** |
| **210511_s_at** | [**inhibin, beta A**](https://david.ncifcrf.gov/geneReportFull.jsp?rowids=825966) | **1,2 (0,93 - 1,56)** | **0.17** | **1,04 (0,87 - 1,24)** | **0.67** | **1,36 (1,11 - 1,67)** | **2.80E-03** | **1,43 (0,94 - 2,18)** | **0.091** |
| **1561042_at** | [**integrin, beta 1 (fibronectin receptor, beta polypeptide, antigen CD29 includes MDF2, MSK12)**](https://david.ncifcrf.gov/geneReportFull.jsp?rowids=784977) | **0,92 (0,65 - 1,29)** | **0.62** | **1,28 (0,99 - 1,65)** | **0.062** | **0,9 (0,66 - 1,23)** | **0.51** | **0,81 (0,5 - 1,31)** | **0.39** |
| **204444_at** | [**kinesin family member 11**](https://david.ncifcrf.gov/geneReportFull.jsp?rowids=809693) | **1,18 (0,91 - 1,53)** | **0.22** | **1,76 (1,47 - 2,11)** | **4.40E-10** | **1,41 (1,15 - 1,73)** | **7.80E-04** | **1,18 (0,78 - 1,79)** | **0.43** |
| **204709_s_at** | [**kinesin family member 23**](https://david.ncifcrf.gov/geneReportFull.jsp?rowids=823458) | **1,3 (1 - 1,68)** | **0.05** | **1,86 (1,56 - 2,23)** | **7.70E-12** | **1,73 (1,41 - 2,12)** | **1.00E-07** | **1,32 (0,87 - 2,01)** | **0.19** |
| **209408_at** | [**kinesin family member 2C**](https://david.ncifcrf.gov/geneReportFull.jsp?rowids=801471) | **0,87 (0,67 - 1,13)** | **0.28** | **1,61 (1,34 - 1,93)** | **1,9 E-07** | **1,3 (1,07 - 1,6)** | **9.80E-03** | **0,78 (0,51 - 1,18)** | **0.24** |
| **209680_s_at** | [**kinesin family member C1**](https://david.ncifcrf.gov/geneReportFull.jsp?rowids=802146) | **1,05 (0,81 - 1,36)** | **0.74** | **1,44 (1,2 - 1,72)** | **6.60E-05** | **1,55 (1,26 - 1,9)** | **2.40E-05** | **0,84 (0,55 - 1,27)** | **0.4** |
| **243831_at** | [**mitogen-activated protein kinase 6**](https://david.ncifcrf.gov/geneReportFull.jsp?rowids=788114) | **0,76 (0,54 - 1,07)** | **0.12** | **0,9 (0,69 - 1,16)** | **0.4** | **0,86 (0,63 - 1,18)** | **0.36** | **1,12 (0,7 - 1,8)** | **0.64** |
| **211449_at** | [**mutS homolog 6 (E. coli)**](https://david.ncifcrf.gov/geneReportFull.jsp?rowids=798813) | **0,88 (0,68 - 1,14)** | **0.34** | **0,84 (0,71 - 1,01)** | **0.059** | **0,71 (0,58 - 0,87)** | **1.10E-03** | **1,07 (0,71 - 1,63)** | **0.74** |
| **218662_s_at** | [**non-SMC condensin I complex, subunit G**](https://david.ncifcrf.gov/geneReportFull.jsp?rowids=793059) | **1,47 (1,13 - 1,91)** | **3.80E-03** | **1,78 (1,48 - 2,13)** | **2.70E-10** | **1,32 (1,08 - 1,62)** | **0.0067** | **1,12 (0,74 - 1,69)** | **0.6** |
| **212949_at** | [**non-SMC condensin I complex, subunit H**](https://david.ncifcrf.gov/geneReportFull.jsp?rowids=803990) | **0,8 (0,61 - 1,04)** | **0.09** | **2 (1,67 - 2,4)** | **2.50E-14** | **1,24 (1,02 - 1,52)** | **0.035** | **1,01 (0,67 - 1,53)** | **0.97** |
| **218039_at** | **NUSAP1 .Nucleolar and spindle associated protein 1** | **1.21 (0.93 − 1.56)** | **0.16** | **2.13 (1.77 − 2.57)** | **2.2E−16** | **1.53 (1.25 − 1.88)** | **3.3e−05** | **0.92 (0.6 − 1.39)** | **0.68** |
| **203554_x_at** | **PTTG1. Pituitary tumor-transforming 1** | **1.39 (1.07 − 1.81)** | **0.013** | **1.95 (1.62 − 2.34)** | **6.3E−13** | **1.26 (1.03 − 1.54)** | **0.027** | **1.07 (0.71 − 1.62)** | **0.74** |
| **218009_s_at** | **PRC1. Protein regulator of cytokinesis 1** | **1.01 (0.78 − 1.31)** | **0.95** | **2.32 (1.93 − 2.8)** | **< 1E−16** | **1.6 (1.3 − 1.96)** | **5.3e−06** | **0.98 (0.65 − 1.49)** | **0.92** |
| **201663_s_at** | **SMC4. Structural maintenance of chromosomes 4-like 1 (yeast)** | **0.96 (0.74 − 1.24)** | **0.75** | **1.33 (1.11 − 1.58)** | **1.70E-03** | **1.19 (0.97 − 1.45)** | **0.095** | **0.76 (0.5 − 1.16)** | **0.2** |
| **237920_at** | [**synaptonemal complex protein 2**](https://david.ncifcrf.gov/geneReportFull.jsp?rowids=824348) | **1,08 (0,77 - 1,51)** | **0.67** | **1,11 (0,86 - 1,43)** | **0.42** | **1,04 (0,76 - 1,43)** | **0.78** | **0,89 (0,55 - 1,43)** | **0.63** |
| **218308_at** | [**transforming, acidic coiled-coil containing protein 3**](https://david.ncifcrf.gov/geneReportFull.jsp?rowids=776047) | **0,93 (0,72 - 1,21)** | **0.59** | **1,8 (1,5 - 2,16)** | **1.20E-10** | **1,13 (0,93 - 1,39)** | **0.22** | **0,77 (0,5 - 1,16)** | **0.21** |
| **240406_at** | [**ubiquitin specific peptidase 16**](https://david.ncifcrf.gov/geneReportFull.jsp?rowids=791042) | **1,22 (0,87 - 1,72)** | **0.24** | **1,2 (0,93 - 1,56)** | **0.16** | **0,75 (0,55 - 1,03)** | **0.074** | **0,95 (0,59 - 1,52)** | **0.82** |
| **202954_at** | **UBE2C. Ubiquitin-conjugating enzyme E2C** | **0.92 (0.71 − 1.19)** | **0.53** | **1.78 (1.48 − 2.13)** | **3.9E−10** | **1.46 (1.19 − 1.79)** | **2.50E-04** | **0.69 (0.46 − 1.06)** | **0.088** |
| **225655_at** | **UHRF1. Ubiquitin-like, containing PHD and RING finger domains, 1** | **1.16 (0.83 − 1.63)** | **0.38** | **1.75 (1.35 − 2.28)** | **2.4E−05** | **1.05 (0.77 − 1.44)** | **0.76** | **1.03 (0.64 − 1.65)** | **0.92** |
| **1554158_at** | [**zinc finger, MYND domain containing 11**](https://david.ncifcrf.gov/geneReportFull.jsp?rowids=818324) | **1,18 (0,85 - 1,66)** | **0.32** | **0,98 (0,76 - 1,26)** | **0.86** | **0,85 (0,62 - 1,16)** | **0.3** | **0,72 (0,45 - 1,16)** | **0.18** |

**SUPLEMENTARY TABLE 1**
