# Supplementary material for: Mitotic read-out genes confer poor outcome in luminal A breast cancer tumors
Source: Oncotarget. 2017 Feb 21;8(13):21733–40. doi: 10.18632/oncotarget.15562 (PMC5400619; doi:10.18632/oncotarget.15562)
Supplement: Supplementary file 3 [file oncotarget-08-21733-s003.doc]

| **Probe Set** | **Gene Name** | **Overall Survival** | | | | | | | |
| --- | --- | --- | --- | --- | --- | --- | --- | --- | --- |
|  |  | **Basal Like** | | **Luminal A** | | **Luminal B** | | **HER2+** | |
|  |  | **HR (Hazard Ratio)** | **Logrank P value** | **HR (Hazard Ratio)** | **Logrank P value** | **HR (Hazard Ratio)** | **Logrank P value** | **HR (Hazard Ratio)** | **Logrank P value** |
| **204170_s_at** | [**CDC28 protein kinase regulatory subunit 2**](https://david.ncifcrf.gov/geneReportFull.jsp?rowids=817048) | **0,58 (0,32 - 1,02)** | **0.057** | **1,84 (1.24 - 2,73)** | **0.002** | **1.11 (0,73 - 1,68)** | **0.62** | **1,62 (0,76 - 3,48)** | **0.21** |
| **204126_s_at** | [**CDC45 cell division cycle 45-like (S. cerevisiae)**](https://david.ncifcrf.gov/geneReportFull.jsp?rowids=813733) | **0,6 (0,34-1,06)** | **0.075** | **1,98 (1,33-2,94)** | **0.00062** | **1,33 (0,87-2,02)** | **0.19** | **1,36 (0,64-2,91)** | **0.42** |
| **222179_at** | [**CDC5 cell division cycle 5-like (S. pombe)**](https://david.ncifcrf.gov/geneReportFull.jsp?rowids=821458) | **0,87 (0,5 - 1,5)** | **0.61** | **0,79 (0,54 -1,16)** | **0.23** | **0,91 (0,6-1,37)** | **0.65** | **0,96 (0,45 - 2,03)** | **0.9** |
| **213008_at** | [**Fanconi anemia, complementation group I**](https://david.ncifcrf.gov/geneReportFull.jsp?rowids=823221) | **0,69 (0,39-1,21)** | **0.19** | **2,32 (1,55-3,47)** | **2.50E-05** | **1,28 (0,84-1,94)** | **0.25** | **1,59 (0,74-3,42)** | **0.24** |
| **204318_s_at** | **GTSE1. G-2 and S-phase expressed 1** | **0,69 (0,39-1,2)** | **0.19** | **3,23 (2,11-4,94)** | **1.20E-08** | **1,1 (0,73-1,67)** | **0.65** | **2 (0,9-4,46)** | **0.084** |
| **213524_s_at** | [**G0/G1switch 2**](https://david.ncifcrf.gov/geneReportFull.jsp?rowids=777021) | **1,04 (0,6-1,81)** | **0.88** | **0,61 (0,41-0,9)** | **0.011** | **1,02 (0,67-1,55)** | **0.94** | **0,92 (0,43-1,97)** | **0.84** |
| **218726_at** | [**Holliday junction recognition protein**](https://david.ncifcrf.gov/geneReportFull.jsp?rowids=824595) | **0,64 (0,37-1,13)** | **0.12** | **2,51 (1,67-3,78)** | **5.20E-06** | **1,55 (1,02-2,35)** | **0.039** | **2,21 (0,99 - 4,92)** | **0.047** |
| **206205_at** | [**M-phase phosphoprotein 9**](https://david.ncifcrf.gov/geneReportFull.jsp?rowids=777293) | **0,98 (0,56-1,71)** | **0.94** | **1,53 (1,04-2,25)** | **0.03** | **1,02 (0,67-1,54)** | **0.93** | **0,92 (0,43-1,97)** | **0.84** |
| **203362_s_at** | **MAD2L1. MAD2 mitotic arrest deficient-like 1 (yeast)** | **0,77 (0,44 - 1,35)** | **0.36** | **1,85 (1,26 - 2,73)** | **1.60E-03** | **1,35 (0,89 - 2,05)** | **0.15** | **1,19 (0,56 - 2,54)** | **0.66** |
| **204162_at** | **KNTC2. Kinetochore associated 2** | **0,58 (0,33 - 1,03)** | **0.061** | **2,01 (1,36 - 2,99)** | **4.00E-04** | **0,9 (0,59 - 1,36)** | **0.6** | **0,52 (0,23 - 1,15)** | **0.1** |
| **204641_at** | [**NIMA (never in mitosis gene a)-related kinase 2**](https://david.ncifcrf.gov/geneReportFull.jsp?rowids=791562) | **0,7 (0,4 - 1,23)** | **0.22** | **1,84 (1,24 - 2,72)** | **1.90E-03** | **1,12 (0,74 - 1,69)** | **0.59** | **2,01 (0,92 - 4,39)** | **0.075** |
| **223381_at** | [**NUF2, NDC80 kinetochore complex component, homolog (S. cerevisiae)**](https://david.ncifcrf.gov/geneReportFull.jsp?rowids=809559) | **0,46 (0,21-1,03)** | **0.054** | **1,26 (0,72-2,22)** | **0.42** | **1,13 (0,54 -2,37)** | **0.75** | **2,46 (0,93-6,47)** | **0.06** |
| **219148_at** | [**PDZ binding kinase**](https://david.ncifcrf.gov/geneReportFull.jsp?rowids=816657) | **0,43 (0,24-0,79)** | **5.00E-03** | **1,74 (1,18-2,57)** | **4.40E-03** | **1,84(1,19-2,82)** | **4.90E-03** | **0,84 (0,39-1,79)** | **0.65** |
| **204558_at** | [**RAD54-like (S. cerevisiae)**](https://david.ncifcrf.gov/geneReportFull.jsp?rowids=799631) | **0,79 (0,45-1,37)** | **0.4** | **1,09 (0,74-1,58)** | **0.67** | **1,16 (0,76-1,76)** | **0.49** | **1,35 (0,63-2,91)** | **0.44** |
| **222077_s_at** | [**Rac GTPase activating protein 1 pseudogene; Rac GTPase activating protein 1**](https://david.ncifcrf.gov/geneReportFull.jsp?rowids=795700) | **0,89 (0,51-1,54)** | **0.67** | **2,77 (1,84-4,19)** | **3.90E-07** | **1,12 (0,74-1,7)** | **0.58** | **1,3 (0,61-2,77)** | **0.5** |
| **235572_at** | [**SPC24, NDC80 kinetochore complex component, homolog (S. cerevisiae)**](https://david.ncifcrf.gov/geneReportFull.jsp?rowids=803549) | **0,66 (0,31-1,42)** | **0.28** | **1,52 (0,85-2,69)** | **0.15** | **1,92 (0,89-4,12)** | **0.089** | **1,56 (0,62-3,89)** | **0.34** |
| **209891_at** | [**SPC25, NDC80 kinetochore complex component, homolog (S. cerevisiae)**](https://david.ncifcrf.gov/geneReportFull.jsp?rowids=821580) | **0,8 (0,46-1,41)** | **0.45** | **2,47 (1,65-3,7)** | **5.90E-06** | **1,23 (0,81-2,87)** | **0.33** | **0,94 (0,44-2,02)** | **0.88** |
| **210052_s_at** | **TPX2. Microtubule-associated protein homolog (Xenopus laevis)** | **0,52 (0,29 - 0,92)** | **0.024** | **2,23 (1,48 - 3,35)** | **7.50E-05** | **1,33 (0,88 - 2,02)** | **0.18** | **1,02 (0,48 - 2,16)** | **0.97** |
| **204822_at** | [**TTK protein kinase**](https://david.ncifcrf.gov/geneReportFull.jsp?rowids=814656) | **0,96 (0,55 – 1,67)** | **0.89** | **3,15 (2,07 – 4,79)** | **1.7E-8** | **1,03 (0,68 - 1,56)** | **0.88** | **1,13 (0,53 - 2,4)** | **0.76** |
| **204026_s_at** | [**ZW10 interactor**](https://david.ncifcrf.gov/geneReportFull.jsp?rowids=783593) | **0,7 (0,4 - 1,23)** | **0.21** | **1,64 (1,12 - 2,42)** | **0.01** | **1,64 (1,07 - 2,51)** | **0.021** | **0,78 (0,36 - 1,68)** | **0.52** |
| **222608_s_at** | **ANLN. Anillin, actin binding protein (scraps homolog, Drosophila)** | **0,91 (0,43 . 1,94)** | **0.81** | **2,68 (1,44 - 4,99)** | **1.20E-03** | **2 (0,92 - 4,35)** | **0.073** | **1,26 (0,51 - 3,11)** | **0.61** |
| **212023_s_at** | **MKI67. Antigen identified by monoclonal antibody Ki-67** | **0,76 (0,44 - 1,33)** | **0.34** | **1,47 (1,01 - 2,16)** | **0.046** | **1,43 (0,94 - 2,18)** | **0.093** | **1,63 (0,74 - 3,56)** | **0.22** |
| **219918_s_at** | **ASPM. Asp (abnormal spindle)-like, microcephaly associated (Drosophila)** | **0,59 (0,33 - 1,04)** | **0.065** | **2,3 (1,55 - 3,43)** | **2.50E-05** | **1,31 (0,86 - 1,99)** | **0.2** | **0,99 (0,46 - 2,1)** | **0.98** |
| **204092_s_at** | [**AURKA. Aurora kinase A; aurora kinase A pseudogene 1**](https://david.ncifcrf.gov/geneReportFull.jsp?rowids=782105) | **0,66 (0,38 - 1,16)** | **0.14** | **2,04 (1,37 - 3,04)** | **3.7E-04** | **1,19 (0,79 - 1,81)** | **0.41** | **0,98 (0,46 - 2,09)** | **0.96** |
| **210334_x_at** | **BIRC5. Baculoviral IAP repeat-containing 5 (survivin)** | **0,6 (0,34 - 1,05)** | **0.07** | **1,58 (1,07 - 2,32)** | **0.02** | **1,28 (0,84 - 1,94)** | **0.25** | **1,02 (0,48 - 2,28)** | **0.96** |
| **209642_at** | **BUB1. Budding uninhibited by benzimidazoles 1 homolog (yeast)** | **0,83 (0,48 -1,45)** | **0.52** | **2,72 (1,81 - 4,09)** | **5.2E-07** | **1,25 (0,82 - 1,89)** | **0.3** | **1,28 (0,6 - 2,73)** | **0.52** |
| **203755_at** | [**budding uninhibited by benzimidazoles 1 homolog beta (yeast)**](https://david.ncifcrf.gov/geneReportFull.jsp?rowids=775002) | **0,45 (0,25 - 0,81)** | **6.40E-03** | **3,04 (2,01 - 4,61)** | **3.9E-08** | **1,38 (0,91 - 2,09)** | **0.13** | **1,1 (0,52 - 2,34)** | **0.81** |
| **210559_s_at** | [**cell division cycle 2, G1 to S and G2 to M**](https://david.ncifcrf.gov/geneReportFull.jsp?rowids=815142) | **0,83 ( 0,47 - 1,44)** | **0.5** | **1,65 (1,12 - 2,43)** | **0.01** | **1,47 (0,97 - 2,25)** | **0.071** | **0,64 (0,29 - 1,39)** | **0.25** |
| **202870_s_at** | **CDC20. Cell division cycle 20 homolog (S. cerevisiae)** | **0,61 (0,35 - 1,06)** | **0.077** | **2,12 (1,42 - 3,18)** | **1.90E-04** | **1,35 (0,89 - 2,06)** | **0.16** | **1,89 (0,86 - 4,13)** | **0.1** |
| **217010_s_at** | [**cell division cycle 25 homolog C (S. pombe)**](https://david.ncifcrf.gov/geneReportFull.jsp?rowids=817790) | **0,84 (0,48 - 1,46)** | **0.54** | **1,5 (1,02 - 2,2)** | **0.037** | **1,5 (0,99 - 2,29)** | **0.055** | **0,53 (0,24 - 1,14)** | **0.099** |
| **223307_at** | **CDCA3. Cell division cycle associated 3** | **0,78 (0,37 - 1,67)** | **0.52** | **2,07 (1,15 - 3,72)** | **1.30E-02** | **1,76 (0,82 - 3,77)** | **0.14** | **1,42 (0,57 - 3,52)** | **0.45** |
| **224753_at** | [**cell division cycle associated 5**](https://david.ncifcrf.gov/geneReportFull.jsp?rowids=789361) | **0,49 (0,22 - 1,1)** | **0.076** | **1,74 (0,98 - 3,09)** | **0.056** | **2,31 (1,04 - 5,1)** | **0.034** | **1,35 (0,54 - 3,33)** | **0.52** |
| **204962_s_at** | **CENPA. Centromere protein A, 17kDa** | **0,45 (0,25 - 0,81)** | **6.20E-03** | **2,37 (1,58 - 3,55)** | **1.50E-05** | **1,23 (0,81 - 1,87)** | **0.34** | **0,81 (0,38 - 1,73)** | **0.58** |
| **205046_at** | [**centromere protein E, 312kDa**](https://david.ncifcrf.gov/geneReportFull.jsp?rowids=789965) | **0,59 (0,34 - 1,05)** | **0.068** | **2,15 (1,45 - 3,2)** | **1.00E-04** | **1,14 (0,76 - 1,73)** | **0.52** | **0,87 (0,41 - 1,85)** | **0.71** |
| **207828_s_at** | **Centromere protein F, 350/400ka (mitosinCENPF. )** | **0,63 (0,36 - 1,11)** | **0.11** | **2,14 (1,44 - 3,19)** | **1.20E-04** | **1,49 (0,98 - 2,28)** | **0.062** | **1,63 (0,76 - 3,52)** | **0.21** |
| **218542_at** | **CEP55. Chromosome 10 open reading frame 3** | **0,59 (0,33 - 1,05)** | **0.071** | **2,8 (1,85 - 4,23)** | **3.50E-07** | **1,16 (0,77 - 1,76)** | **0.48** | **1,36 (0,64 - 2,92)** | **0.42** |
| **228868_x_at** | [**chromatin licensing and DNA replication factor 1**](https://david.ncifcrf.gov/geneReportFull.jsp?rowids=780175) | **1,3 (0,61 - 2,78)** | **0.49** | **1,56 (0,88 - 2,77)** | **0.13** | **1,54 (0,73 - 3,25)** | **0.26** | **0,8 (0,32 - 1,99)** | **0.63** |
| **227165_at** | [**chromosome 13 open reading frame 3**](https://david.ncifcrf.gov/geneReportFull.jsp?rowids=809850) | **0,89 (0,42 - 1,9)** | **0.76** | **1,5 (0,85 - 2,64)** | **0.16** | **3,22 (1,41 - 7,32)** | **3.20E-03** | **1,24 (0,5 - 3,04)** | **0.64** |
| **244173_at** | [**chromosome 14 open reading frame 106**](https://david.ncifcrf.gov/geneReportFull.jsp?rowids=793643) | **0,98 (0,46 - 2,09)** | **0.95** | **1,2 (0,69 - 2,11)** | **0.52** | **1,55 (0,72 - 3,36)** | **0.26** | **0,61 (0,25 - 1,53)** | **0.29** |
| **217640_x_at** | [**chromosome 18 open reading frame 24**](https://david.ncifcrf.gov/geneReportFull.jsp?rowids=773124) | **0,47 (0,26 - 0,84)** | **9.50E-03** | **1,6 (1,09 - 2,36)** | **0.016** | **0,81 (0,54 - 1,23)** | **0.33** | **0,92 (0,43 - 1,97)** | **0.84** |
| **203418_at** | **CCNA2. Cyclin A2** | **0,76 (0,44 - 1,33)** | **0.34** | **1,65 (1,12 - 2,42)** | **0.01** | **1,51 (0,99 - 2,29)** | **0.055** | **1,79 (0,83 - 3,86)** | **0.13** |
| **214710_s_at** | **CCNB1. Cyclin B1** | **0,65 (0,37 - 1,15)** | **0.14** | **1,57 (1,07 - 2,32)** | **0.021** | **1,77 (1,16 - 2,72)** | **7.90E-03** | **0,8 (0,37 - 1,71)** | **0.56** |
| 202705_at | [**cyclin B2**](https://david.ncifcrf.gov/geneReportFull.jsp?rowids=813528) | **0,44 (0,24 - 0,8)** | **5.50E-03** | **2,68 (1,77 - 4,05)** | **1.2E-06** | **1,45 (0,95 - 2,21)** | **0.083** | **1,28 (0,6 - 2,73)** | **0.52** |
| 205034_at | **CCNE2 Cyclin E2** | **0,52 (0,29 - 0,94)** | **0.027** | **2,3 (1,54 - 3,42)** | **2.60E-05** | **1,01 (0,67 - 1,53)** | **0.95** | **2,19 (1 - 4,81)** | **0.045** |
| 209714_s_at | [**cyclin-dependent kinase inhibitor 3**](https://david.ncifcrf.gov/geneReportFull.jsp?rowids=815446) | **1,21 (0,7 - 2,1)** | **0.49** | **1,96 (1,32 - 2,93)** | **7.10E-04** | **1,34 (0,89 - 2,04)** | **0.16** | **0,9 (0,42 - 1,92)** | **0.79** |
| 238048_at | [**cytoplasmic linker associated protein 2**](https://david.ncifcrf.gov/geneReportFull.jsp?rowids=794156) | **0,65 (0,3 - 1,39)** | **0.26** | **0,94 (0,54 - 1,66)** | **0.84** | **1,33 (0,63 - 2,84)** | **0.45** | **1,77 (0,7 - 4,51)** | **0.22** |
| 203764_at | **DLGAP5. Discs, large homolog 7 (Drosophila)** | **0,74 (0,42 - 1,31)** | **0.3** | **2,46 (1,63 - 3,7)** | **8.80E-06** | **1,06 (0,7 - 1,6)** | **0.78** | **0,5 (0,22 - 1,11)** | **0.08** |
| 204455_at | **DST. Dystonin** | **1,93 (1,09 - 3,4)** | **0.022** | **0,66 (0,45 - 0,96)** | **0.03** | **0,79 (0,52 - 1,2)** | **0.27** | **0,97 (0,46 - 2,07)** | **0.94** |
| 201983_s_at | [**epidermal growth factor receptor (erythroblastic leukemia viral (v-erb-b) oncogene homolog, avian)**](https://david.ncifcrf.gov/geneReportFull.jsp?rowids=816368) | **1,64 (0,93 - 2,87(** | **0.084** | **0,89 (0,61 - 1,29)** | **0.53** | **0,66 (0,43 - 1,01)** | **0.052** | **1,38 (0,64 - 2,97)** | **0.41** |
| 219650_at | [**excision repair cross-complementing rodent repair deficiency, complementation group 6-like**](https://david.ncifcrf.gov/geneReportFull.jsp?rowids=820324) | **0,83 (0,48 - 1,43)** | **0.5** | **1,63 (1,1 - 2,41)** | **0.013** | **1,16 (0,77 - 1,76)** | **0.48** | **1,29 (0,6 - 2,76)** | **0.51** |
| 204603_at | [**exonuclease 1**](https://david.ncifcrf.gov/geneReportFull.jsp?rowids=776417) | **0,67 (0,38 - 1,17)** | **0.16** | **1,16 (0,79 - 1,69)** | **0.44** | **1,41 (0,93 - 2,13)** | **0.11** | **0,92 (0,43 - 1,96)** | **0.83** |
| 204817_at | [**extra spindle pole bodies homolog 1 (S. cerevisiae)**](https://david.ncifcrf.gov/geneReportFull.jsp?rowids=802581) | **0,43 (0,24 - 0,77)** | **3.30E-03** | **2,19 (1,47 - 3,26)** | **7.80E-05** | **1,18 (0,97 - 1,45)** | **0.1** | **1,21 (0,57 - 2,59)** | **0.62** |
| 225687_at | **FAM83D. Chromosome 20 open reading frame 129** | **0,98 (0,46 - 2,1)** | **0.96** | **2,56 (1,4 - 4,7)** | **1.70E-03** | **1,35 (0,64 - 2,86)** | **0.43** | **1,55 (0,62 - 3,87)** | **0.34** |
| 202580_x_at | **FOXM1. Forkhead box M1** | **0,49 (0,27 - 0,87)** | **0.014** | **2,63 (1,74 - 4)** | **2.30E-06** | **1,63 (1,06 - 2,5)** | **0.025** | **0,93 (0,44 - 1,99)** | **0.86** |
| 204456_s_at | [**growth arrest-specific 1**](https://david.ncifcrf.gov/geneReportFull.jsp?rowids=794296) | **0,91 (0,52 - 1,58)** | **0.74** | **0,99 (0,68 - 1,45)** | **0.98** | **1,1 (0,73 - 1,67)** | **0.64** | **0,36 (0,16 - 0,84)** | **0.013** |
| 235709_at | [**growth arrest-specific 2 like 3**](https://david.ncifcrf.gov/geneReportFull.jsp?rowids=787351) | **0,69 (0,32 - 1,52)** | **0.36** | **0,93 (0,53 - 1,64)** | **0.81** | **1,11 (0,53 - 2,34)** | **0.78** | **0,71 (0,29 - 1,77)** | **0.46** |
| 210511_s_at | [**inhibin, beta A**](https://david.ncifcrf.gov/geneReportFull.jsp?rowids=825966) | **1,25 (0,72 - 2,17)** | **0.43** | **0,89 (0,61 - 1,3)** | **0.54** | **1,59 (1,04 - 2,43)** | **0.03** | **1,25 (0,58 - 2,67)** | **0.56** |
| 1561042_at | [**integrin, beta 1 (fibronectin receptor, beta polypeptide, antigen CD29 includes MDF2, MSK12)**](https://david.ncifcrf.gov/geneReportFull.jsp?rowids=784977) | **1,49 (0,7 - 3,2)** | **0.3** | **2,4 (1,32 - 4,37)** | **3.00E-03** | **1,1 (0,52 - 2,3)** | **0.81** | **0,93 (0,38 - 2,3)** | **0.88** |
| 204444_at | [**kinesin family member 11**](https://david.ncifcrf.gov/geneReportFull.jsp?rowids=809693) | **0,76 (0,44 - 1,33)** | **0.34** | **2,29 (1,54 - 3,41)** | **2.80E-05** | **1,39 (0,92 - 2,11)** | **0.12** | **1,47 (0,68 - 3,14)** | **0.32** |
| 204709_s_at | [**kinesin family member 23**](https://david.ncifcrf.gov/geneReportFull.jsp?rowids=823458) | **0,78 (0,45 - 1,38)** | **0.4** | **1,79 (1,22 - 2,64)** | **2.70E-03** | **1,43 (0,94 - 2,18)** | **0.097** | **0,91 (0,43 - 1,85)** | **0.82** |
| 209408_at | [**kinesin family member 2C**](https://david.ncifcrf.gov/geneReportFull.jsp?rowids=801471) | **0,49 (0,28 - 0,87)** | **0.012** | **2,1 (1,41 - 3,12)** | **1.80E-04** | **1,42 (0,94 - 2,16)** | **0.097** | **1,64 (0,76 - 3,53)** | **0.21** |
| 209680_s_at | [**kinesin family member C1**](https://david.ncifcrf.gov/geneReportFull.jsp?rowids=802146) | **0,76 (0,44 - 1,33)** | **0.34** | **1,45 (0,99 - 2,12)** | **0.057** | **1,13 (0,74 - 1,71)** | **0.57** | **0,89 (0,42 - 1,9)** | **0.76** |
| 243831_at | [**mitogen-activated protein kinase 6**](https://david.ncifcrf.gov/geneReportFull.jsp?rowids=788114) | **0,4 (0,18 - 0,89)** | **0.02** | **0,96 (0,54 - 1,69)** | **0.89** | **1,22 (0,57 - 2,59)** | **0.61** | **0,73 (0,3 - 1,82)** | **0.5** |
| 211449_at | [**mutS homolog 6 (E. coli)**](https://david.ncifcrf.gov/geneReportFull.jsp?rowids=798813) | **0,84 (0,49 - 1,47)** | **0.55** | **0,9 (0,62 - 1,32)** | **0.59** | **1,07 (0,71 - 1,63)** | **0.73** | **2,22 (1 - 4,93)** | **0.046** |
| 218662_s_at | [**non-SMC condensin I complex, subunit G**](https://david.ncifcrf.gov/geneReportFull.jsp?rowids=793059) | **0,76 (0,44 - 1,33)** | **0.34** | **1,89 (1,28 - 2,81)** | **1.20E-03** | **1,28 (0,84 - 1,95)** | **0.24** | **1,05 (0,49 - 2,24)** | **0.89** |
| 212949_at | [**non-SMC condensin I complex, subunit H**](https://david.ncifcrf.gov/geneReportFull.jsp?rowids=803990) | **0,4 (0,22 - 0,73)** | **2.00E-03** | **1,91 (1,3 - 2,82)** | **9.00E-04** | **1,32 (0,87 - 2)** | **0.19** | **1,05 (0,5 - 2,24)** | **0.89** |
| 218039_at | **NUSAP1 .Nucleolar and spindle associated protein 1** | **0,63 (0,36 - 1,11)** | **0.1** | **2,91 (1,91 - 4,43)** | **2.00E-07** | **1,27 (0,84 - 1,93)** | **0.26** | **0,85 (0,4 - 1,81)** | **0.67** |
| 203554_x_at | **PTTG1. Pituitary tumor-transforming 1** | **0,89 (0,51 - 1,55)** | **0.68** | **1,76 (1,19 - 2,61)** | **4.50E-03** | **1,26 (0,83 - 1,91)** | **0.29** | **0,81 (0,38 - 1,74)** | **0.59** |
| 218009_s_at | **PRC1. Protein regulator of cytokinesis 1** | **0,77 (0,44 - 1,35)** | **0.36** | **2,64 (1,75 - 3,97)** | **1.50E-06** | **1,61 (1,05 - 2,46)** | **0.027** | **1,35 (0,63 - 2,87)** | **0.44** |
| 201663_s_at | **SMC4. Structural maintenance of chromosomes 4-like 1 (yeast)** | **0,58 (0,33 - 1,03)** | **0.061** | **1,81 (1,23 -2,67)** | **2.40E-03** | **1,25 (0,83 - 1,9)** | **0.28** | **0,96 (0,45 - 2,05)** | **0.92** |
| 237920_at | [**synaptonemal complex protein 2**](https://david.ncifcrf.gov/geneReportFull.jsp?rowids=824348) | **0,69 (0,31 - 1,5)** | **0.34** | **1,09 (0,62 - 1,92)** | **0.77** | **0,91 (0,43 - 1,9)** | **0.79** | **1,45 (0,59 - 3,57)** | **0.42** |
| 218308_at | [**transforming, acidic coiled-coil containing protein 3**](https://david.ncifcrf.gov/geneReportFull.jsp?rowids=776047) | **0,51 (0,29 - 0,9)** | **0.019** | **2,03 (1,37 - 3,01)** | **3.40E-04** | **0,95 (0,63 - 1,43)** | **0.8** | **1,23 (0,57 - 2,63)** | **0.6** |
| 240406_at | [**ubiquitin specific peptidase 16**](https://david.ncifcrf.gov/geneReportFull.jsp?rowids=791042) | **1,55 (0,72 - 3,35)** | **0.26** | **1,13 (0,64 - 1,98)** | **0.67** | **1,14 (0,54 -2,4)** | **0.73** | **2,07 (0,79 - 5,47)** | **0.13** |
| 202954_at | **UBE2C. Ubiquitin-conjugating enzyme E2C** | **0,72 (0,41 - 1,25)** | **0.74** | **1,76 (1,19 - 2,6)** | **4.20E-03** | **1,71 (1,11 - 2,61)** | **0.013** | **0,9 (0,42 - 1,92)** | **0.79** |
| 225655_at | **UHRF1. Ubiquitin-like, containing PHD and RING finger domains, 1** | **0,58 (0,27 - 1,27)** | **0.17** | **0,95 (0,54 - 1,66)** | **0.85** | **1,17 (0,56 - 2,46)** | **0.68** | **1,87 (0,73 - 7,74)** | **0.18** |
| 1554158_at | [**zinc finger, MYND domain containing 11**](https://david.ncifcrf.gov/geneReportFull.jsp?rowids=818324) | **1,39 (0,65 - 2,98)** | **0.39** | **0,79 (0,45 - 1,39)** | **0.42** | **0,68 (0,32 - 1,46)** | **0.33** | **0,54 (0,2 - 1,41)** | **0.2** |

**SUPLEMENTARY TABLE 2**
